# Leveraging ICT Tools to Improve Kidney Health: A Comprehensive Review of Innovations in Nephrology

**DOI:** 10.3390/healthcare14060785

**Published:** 2026-03-20

**Authors:** Abel Mata-Lima, José Javier Serrano-Olmedo, Ana Rita Paquete

**Affiliations:** 1 Center for Biomedical Technology (CTB), Universidad Politécnica de Madrid (UPM), 28040 Madrid, Spain; 2 Centro de Investigación Biomédica en Red Para Bioengeniería, Biomateriales y Nanomedicina, Instituto de Salud Carlos III, 28029 Madrid, Spain; 3 Renal Division, Hospital Divino Espirito Santo (HDES), 9500-370 Ponta Delgada, Portugal

**Keywords:** nephrology, information and communication technology, remote patient monitoring, artificial intelligence, narrative review

## Abstract

**Background:** Chronic kidney disease (CKD) and end-stage renal disease (ESRD) represent a growing global health burden, affecting nearly one in ten adults worldwide. CKD is associated with high morbidity, premature mortality, reduced quality of life and enormous healthcare costs, and is primarily driven by dialysis and kidney transplantation. The silent and progressive nature of CKD means that most patients are diagnosed late, when irreversible damage has already occurred and costly kidney replacement therapies (KRT) become necessary. Dialysis services are resource-intensive, requiring significant infrastructure, specialized staff, and consumables, which makes them especially challenging to sustain in low- and middle-income countries. Traditional models of nephrology, care center-based dialysis and fragmented follow-up are increasingly inadequate in meeting the demands of a rising CKD population. These challenges highlight the urgent need for innovative approaches that enhance efficiency, improve patient outcomes, and expand access. **Objective:** This review aims to analyze the current landscape of information and communication technology (ICT) applications in nephrology and to evaluate how digital innovations are reconfiguring kidney therapy. Specifically, it seeks to identify the major ICT tools that are currently in use, assess their clinical and operational impact, and discuss their role in creating more sustainable, patient-centered kidney care models. This study reviews and analyzes ICT tools that are reconfiguring nephrology, including remote monitoring, AI, wearables, patient engagement apps and data dashboards. **Methods:** Narrative and scoping review of recent innovations in nephrology, including remote patient monitoring (RPM), telehealth, artificial intelligence (AI) analytics, wearable sensors, and clinical decision support platforms. **Results:** ICT tools such as Sharesource, Versia, telenephrology platforms, medical assistant for Chronic Care Service (MACCS), AI-based predictive analytics, wearable devices and patient engagement apps have improved patient outcomes, adherence, and early detection of complications. Key metrics include technique survival, hospitalization rate, patient-reported outcomes, workflow efficiency, and prediction accuracy. The relevant literature describing the potential of digital health technologies, including ICT platforms, artificial intelligence tools, and remote monitoring systems, to transform nephrology care was retrieved and screened for inclusion in this narrative review. **Conclusions:** ICT has shifted nephrology from reactive to proactive care, enhancing accessibility, patient empowerment and clinical efficiency. Future directions include precision nephrology, fully wearable kidneys, AI integration and large language models for education and triage. Challenges include digital divide, regulatory heterogeneity, cost and the need for long-term evidence.

## 1. Introduction

CKD represents a major and growing global public health challenge, affecting more than 850 million people worldwide and accounting for a substantial proportion of cardiovascular morbidity, premature mortality, and healthcare expenditure [1,2]. The rising prevalence of CKD is largely driven by aging populations and the increasing burden of diabetes, hypertension, and metabolic disease [2,3]. CKD is typically progressive and irreversible, often remaining clinically silent until advanced stages, when therapeutic options are limited and costly kidney replacement therapies, dialysis or kidney transplantation, become necessary [4,5]. Consequently, health systems face mounting pressure to deliver kidney care that is not only clinically effective but also sustainable and equitable.

CKD is a progressive condition characterized by persistent abnormalities in kidney structure or function lasting at least three months, with implications for health. The prevalence of ESRD and the demand for KRT continue to increase globally, placing substantial clinical and economic pressure on healthcare systems [6,7]. Traditional models of nephrology care have been predominantly reactive, characterized by episodic outpatient visits, delayed referrals, and late-stage interventions [3]. Such approaches have proven insufficient to halt disease progression, prevent complications, or adequately support patients across the continuum of CKD, end-stage kidney disease (ESKD) and post-transplant care. Moreover, access to nephrology services remains uneven, with marked disparities in rural regions and low- and middle-income countries, further amplifying inequities in kidney patients’ outcomes [4].

In recent years, digital nephrology, defined as the application of ICT, digital health tools and AI to kidney care, has emerged as a transformative paradigm. Telemedicine and remote patient monitoring have enabled the continuity of care beyond traditional clinical settings, allowing for real-time assessment of dialysis adequacy, fluid status, adherence, and early complications [5,8]. These technologies have been particularly impactful in home-based dialysis and transplant follow-up, where timely interventions can reduce hospitalizations and improve technique and graft survival [8].

**Figure 1:**
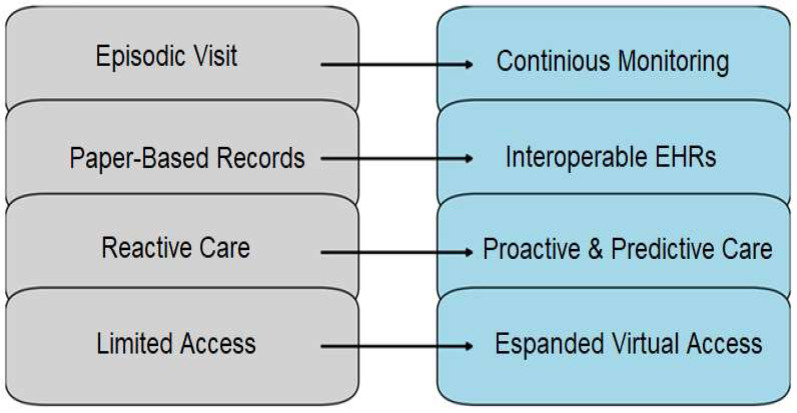
Digital transformation model (traditional vs. digital nephrology).

Kidney disease management is entering a digital era, where information and communication technology (ICT) tools increasingly mediate interactions between patients, clinicians, and healthcare systems [9,10]. Unlike the incremental advancements of the past, digital nephrology represents a true paradigm shift, harnessing mobile health, artificial intelligence (AI), wearable technology and telemedicine to transform the prevention, monitoring and treatment of kidney disease [11,12,13]. In summary, the ICT intervention is a meaningful innovation (see Figure 1).

At the same time, advances in artificial intelligence and machine learning have opened up new opportunities for risk stratification, prediction, and personalization in nephrology. AI-based models can estimate the course of CKD, identify individuals who are at high risk of rapid progression or acute kidney injury, and support earlier, more targeted clinical decision-making [8,9]. Image-based AI applications have improved the interpretation of renal biopsies and retinal images, increasing diagnostic accuracy and reproducibility while reducing inter-observer variability [10]. In transplantation, emerging AI tools show promise in the early detection of rejection and the optimization of immunosuppressive strategies [11].

Together, these digital innovations support a paradigm shift toward precision nephrology, in which continuous data streams, predictive analytics and patient engagement tools enable proactive and individualized care [14,15,16]. However, despite their promise, the integration of digital health and AI into routine nephrology practice remains uneven. Evidence is heterogeneous, long-term outcome data are limited for some technologies, and challenges related to data privacy, regulatory oversight, reimbursement and digital equity persist [17,18]. In this context, a summary of the current evidence is needed.

This narrative review explores the transformative role of ICT, with a focus on AI-based tools in nephrology, and examines how they redefine care models, patient engagement, and clinical outcomes. Special attention was given to the existing limitations and future directions toward sustainable patient-centered kidney care [19,20,21]. The methodology is described below, including information on the databases, keywords, inclusion and exclusion criteria (see Table 1) and synthesis in this investigation.

**Figure 2:**
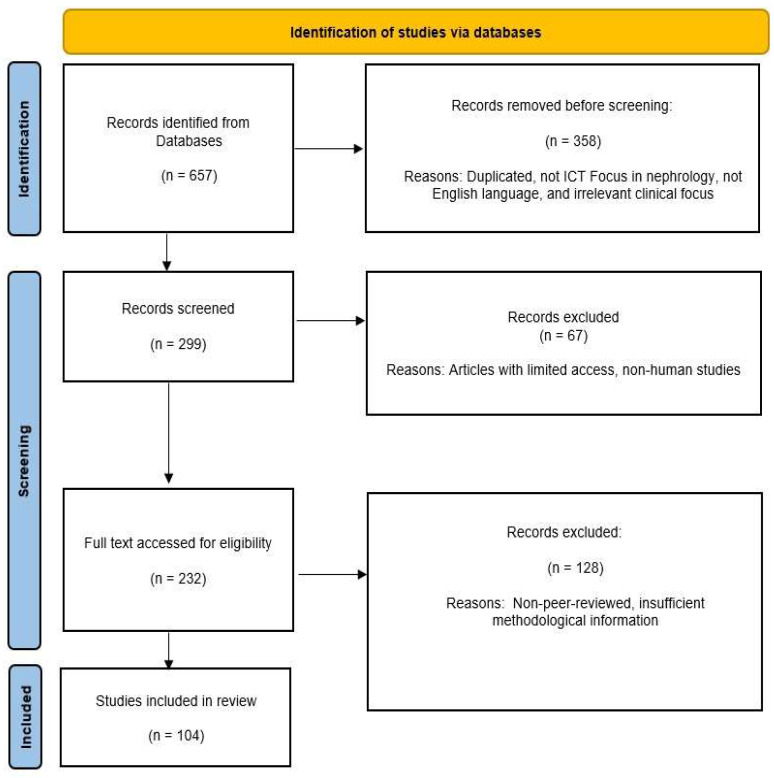
Flow diagram of narrative review of literature.

**Figure 3:**
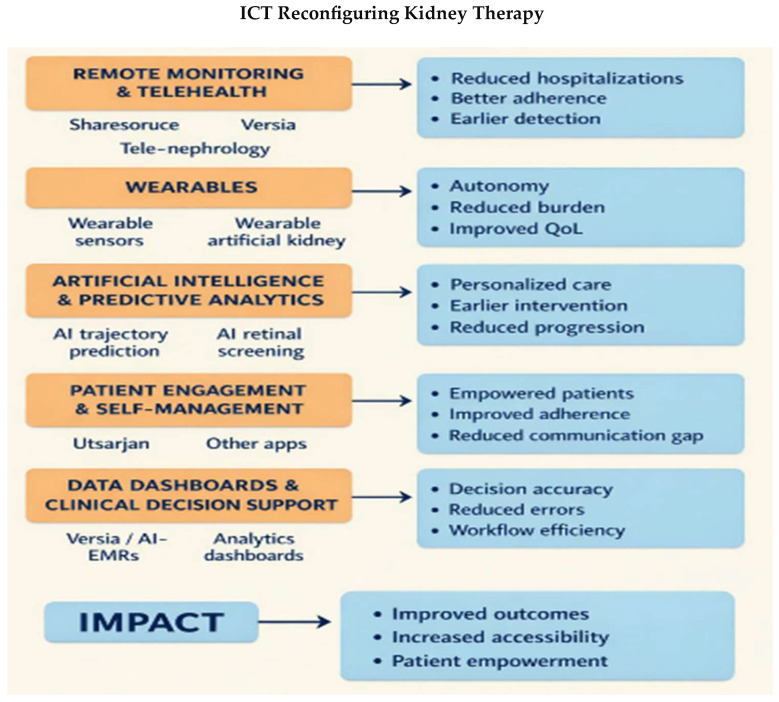
ICT reconfiguring kidney therapy.

The methodology is described in the following section and includes details about the databases searched, the keywords used, and the inclusion and exclusion criteria applied (Table 1), as well as the approach used for the synthesis of evidence. The conceptual model illustrating the transition from traditional to digital nephrology is presented in Figure 1. The Figure 2 shows the flow diagram of record screening. Figure 3 and Table 2 summarize how ICT and AI are influencing renal therapy. Table 3 provides an overview of the ICT tools that are currently used in nephrology and renal care. The Table 4 provides example of vendors os study and implementation of ICT toools, while Table 5 describe their reported clinical impact and Table 6 shows key outcomes and the maturity of ICT tools.

Despite the growing integration of ICT in nephrology care, there are still significant gaps in the existing literature. The current evidence is predominantly derived from pilot studies, small cohorts, or short-term evaluations, limiting conclusions about long-term clinical outcomes and sustainability. Robust health economic analyses assessing the cost-effectiveness and system-level impact are scarce. Methodological heterogeneity across studies further limits the comparability and synthesis of evidence. Patient-centered dimensions, including usability, adherence, digital literacy, and equity implications, are insufficiently explored. Furthermore, few studies address real-world implementation, interoperability with electronic health record systems, and organizational readiness. Ethical and regulatory considerations—particularly those related to data governance, privacy, and algorithmic bias in artificial intelligence applications—remain under-examined. External validation of predictive models in diverse populations is also limited. These gaps underscore the need for rigorous, standardized, and implementation-oriented research to better define the clinical and organizational implications of adopting ICT in nephrology.

## 2. Methodological Approach

Methods: We conducted a narrative/scoping review of the literature that was focused on ICT innovations that were relevant to nephrology and kidney therapy. Major databases (PubMed, Scopus, Web of Science) were searched in December 2025 and January 2026 for articles published from September 2011 to December 2025. Key search terms included: “nephrology,” “kidney disease,” “dialysis,” “renal replacement therapy” “telemedicine,” “remote patient monitoring,” “artificial intelligence,” “machine learning,” “wearables,” “digital health,” and “information systems,” and were combined using Boolean operators. Eligibility was restricted to studies published in English. The review prioritized tools with demonstrated clinical utility or ongoing deployment in kidney care, including remote patient monitoring (RPM) systems, telehealth platforms, AI applications, wearable sensors, and connected dialysis technologies.

This narrative review emphasizes conceptual depth and thematic exploration to illustrate how ICT tools are transforming nephrology.

Using key search terms to identify study titles related to ICT-based tools used in nephrology and renal care (both clinical and experimental), we identified or retrieved 457 records from the PubMed, Web of Science, and Scopus databases.

After the titles and abstract were screened, we excluded 58 records and assessed the full text of 299 records. Finally, 104 articles were included in the narrative review (see Figure 2).

Table 1 provides a structured overview of the criteria for including or excluding studies in a review or for studies focused on AI and ICT Renal Care.

### 2.1. Study Selection

A rigorous approach was employed for study selection and data extraction to ensure reproducibility and the generation of reliable evidence. Initially, titles and abstracts were screened for relevance. In summary, the selection process involved screening titles and abstracts for relevance. The preliminary search includes conducting a basic search in selected databases with applied filters, followed by exporting the results to reference management tools to remove duplicates. Full-text manuscripts were evaluated for inclusion based on their alignment with the study’s objectives. Studies were included if they provided significant insights into AI applications and ICT intervention in nephrology. The screening was performed by three reviewers. Based on this premise, we included 100 articles in this review.

### 2.2. Main Body (Results/Review of Tools)

#### Thematic Review

1. Telenephrology and virtual care

Telemedicine has expanded nephrology services beyond traditional hospital settings [1,22]. Virtual consultations reduce travel burdens for patients on dialysis, enable remote follow-up for transplant recipients, and extend nephrology expertise to underserved regions [22]. These shifts fundamentally change the geography of kidney care.

2. Mobile health applications and patient empowerment

Mobile apps for CKD and dialysis management support self-care by tracking lab results, medications, and lifestyle behaviors [23,24]. Empowering patients through digital literacy and real-time feedback represents a revolution in the traditional patient–provider dynamic, positioning patients as active participants in their care [25,26].

3. Artificial intelligence and predictive analytics

Machine learning models can predict adverse outcomes such as acute kidney injury or graft rejection with high accuracy [27]. ICT-driven predictive analytics enables early intervention, moving the nephrology from reactive to proactive, and potentially transforming outcomes at both individual and population levels [28,29,30].

4. Wearable and sensor technologies

Smart wearables capable of monitoring hydration status, blood pressure, or dialysis adequacy offer continuous, non-invasive insights into patient health [31]. These innovations shift kidney disease management from episodic clinical encounters to continuous digital surveillance.

5. Integration of digital platforms into health systems

The transformative power of ICT lies not only in isolated tools but also in their integration into electronic health records and multi-disciplinary care pathways [32]. Interoperability and real-time data sharing are critical for realizing the full revolutionary potential [12,33] of digital nephrology.

6. Challenges to reconfiguring kidney care

Digital divides, cybersecurity risks, and resistance to technological adoption remain barriers. Furthermore, the pace of innovation often outstrips regulatory and reimbursement frameworks, creating uncertainty for widespread adoption [21,34].

7. Several ICT tools have begun to reshape nephrology practice

- Remote patient monitoring (RPM): Cloud-based platforms such as Sharesource^®^ (Vantive/Baxter) enable continuous monitoring of peritoneal dialysis patients, transmitting treatment data to clinicians in real time [32,33]. This allows for early detection of complications, personalized adjustments, and reduction in hospital visits.
- Versia^®^ (Vantive/Baxter) digital health solutions: Emerging platforms provide patient dashboards, clinician portals, and integration with wearable devices, supporting comprehensive CKD management and engagement in home-based therapy [32].
- Telehealth: Video consultations and digital follow-up systems have become integral in nephrology, particularly during the COVID-19 pandemic [5,15,17]. They have improved access for rural and underserved populations, reduced travel burdens, and facilitated the continuity of care [17].
- Artificial intelligence: AI algorithms are being applied to predict CKD progression, optimize dialysis prescriptions, and support decision-making in kidney transplantation [35]. Machine learning (ML) also enhances pathology interpretation, such as automated biopsy image analysis, with the potential to improve diagnostic accuracy and efficiency [36,37].
- Wearable devices and sensors: Devices that track blood pressure, fluid status and physical activity are increasingly being integrated into nephrology care [37]. Coupled with mobile health applications, they empower patients to actively participate in disease management and provide clinicians with real-time data for early intervention [38,39].

Collectively, these tools demonstrate measurable improvements in patient engagement, efficiency of care delivery and, in some cases, clinical outcomes such as reduced hospitalization rates and better treatment adherence. However, the evidence quality is variable, and many innovations remain in pilot or early adoption stages [40].

Figure 2 and Figure 3 and Table 2, Table 3, Table 4, Table 5 and Table 6 give us:

- A framework diagram to anchor the review.
- A summary table of ICT applications.
- A challenge/solution table to make support for the conclusion actionable.
- Wearable devices and sensors.

Ultrasound plays a key role in modern nephrology workflows, especially in the assessment of acute kidney injury (AKI) and its differentiation from CKD. As a safe, noninvasive, and repeatable modality, ultrasound is widely used as a first-line diagnostic tool because it provides information about renal anatomy, urinary tract obstruction, and renal perfusion. The growing adoption of point-of-care ultrasound and portable imaging devices allows for integration with digital nephrology workflows, including connectivity with electronic health records and clinical decision support systems. On the other hand, imaging techniques such as computed tomography and magnetic resonance imaging may be limited in patients with renal impairment, due to possible contrast-related nephrotoxicity. Ultrasound also facilitates the technological management of vascular access, as it allows for real-time guidance for catheter placement and monitoring of dialysis access [41].

### 2.3. Section A—Remote Monitoring and Telehealth

#### 2.3.1. Remote Monitoring and Telehealth

The integration of remote monitoring (RM) and telehealth platforms into nephrology has transformed the way that patients with CKDESRD and transplant recipients are managed [8,14,17]. These technologies enable continuous data collection, remote consultations, and earlier clinical interventions, addressing the traditional challenges of limited access and late detection of complications [42].

#### 2.3.2. Sharesource (Vantive/Baxter)—Remote Patient Monitoring for Automated Peritoneal Dialysis (APD)

Sharesource is a cloud-based platform developed by Vantive/Baxter that allows clinicians to remotely monitor patients undergoing APD [32]. Through automated data transmission from cyclers, Sharesource provides detailed information on treatment adherence, ultrafiltration, dwell times, and potential alarms [32,33].

RPM using the Sharesource platform increased the adherence rate in APD treatment, and improved the level of serum potassium and the inflammation status [33]. Other studies concluded that the APP tool allows the users to manage their treatments [43].

- Impact: Clinical studies demonstrate that Sharesource reduces the need for unplanned hospital visits, supports technique survival in peritoneal dialysis, and improves patient adherence by enabling real-time troubleshooting [32,33].
- Metrics: Improved technique survival, decreased hospitalization rates, fewer emergency visits, and enhanced patient satisfaction [32,33].

#### 2.3.3. Versia—Renal Data Management System

Versia is a digital ecosystem that consolidates patient data across dialysis modalities. It provides a comprehensive overview of treatment parameters, laboratory results, and clinical events [32].

- Impact: Facilitates personalized care planning and quality benchmarking across dialysis centers, improving clinical decision-making and operational efficiency [32].
- Metrics: Better resource utilization, enhanced workflow efficiency, and support for adherence to quality-of-care metrics [32].

#### 2.3.4. Telenephrology Platforms

Telenephrology platforms allow nephrologists to provide consultations to patients in remote or underserved areas, expanding access to specialized care [44]. Applications range from pre-dialysis management and CKD prevention to follow-up care for transplant and dialysis patients [45,46,47].

- Impact: Improved accessibility in rural regions, reduced travel burden, higher patient engagement, and evidence of equivalent or superior outcomes compared to in-person visits.
- Metrics: Increased patient satisfaction scores, reduced no-show rates, improved blood pressure control, and cost savings through reduced travel and time [45,46,47].

#### 2.3.5. MACCS Platform—Post-Transplant Patient Home Monitoring

The MACCS (Medical Assistant for Chronic Care Support/Service) platform is designed for transplant recipients, offering tools for medication reminders, monitoring of vital parameters, and early detection of complications such as infection or rejection [47].

- Impact: Early detection of adverse events reduces the risk of graft loss and hospital admissions while empowering patients in self-management [47].
- Metrics: Improved graft survival rates, reduced readmission frequency, and increased adherence to immunosuppressive therapy [47].

#### 2.3.6. Overall Impact of Remote Monitoring and Telehealth

Evidence consistently shows that remote monitoring (RM) and telehealth tools enhance the quality of kidney care by enabling proactive interventions [43,48,49].

- Reduced hospitalizations through early detection of complications.
- Better adherence to treatment regimens and prescriptions.
- Earlier detection of adverse events (fluid overload, nonadherence, infections).
- Improved technique survival for dialysis modalities.
- Optimized resource utilization by reducing unnecessary in-person visits.
- Increased patient satisfaction due to convenience, empowerment, and personalization of care.

Metrics: technique survival, hospitalization rate, resource utilization, and patient satisfaction.

### 2.4. Section B—Wearable Devices and Portable Dialysis

#### 2.4.1. Wearables and Portable Kidney Therapies

The integration of wearable technologies into nephrology reflects a paradigm shift from episodic, in-center monitoring to continuous, patient-centered care [31,38]. These tools range from simple biometric sensors to experimental wearable artificial kidneys, each designed to reduce treatment burden [39,40], enhance patient autonomy, and improve quality of life.

#### 2.4.2. Wearable Sensors for Physiological Monitoring

Advances in miniaturized, non-invasive sensors have enabled real-time monitoring of parameters that are critical to kidney health [31,39]. Current devices include:

- Blood pressure monitors (ambulatory and wrist-worn) for detection of hypertension, a major CKD risk factor.
- Hydration sensors using bioimpedance or photoplethysmography, supporting fluid balance management in dialysis patients.
- Glucose sensors (continuous glucose monitoring, (CGM)), which are particularly relevant for patients with diabetic kidney disease.

Impact: Wearable sensors provide continuous data streams that can be integrated with electronic health records and AI-based platforms to enable the proactive management of CKD and dialysis complications. They reduce reliance on clinic-based monitoring and allow for earlier interventions [31,39].

Metrics: Improved blood pressure control, reduced intradialytic hypotension episodes, better ultrafiltration precision, enhanced self-management, and increased patient satisfaction [31,39].

#### 2.4.3. Wearable Artificial Kidneys (WAKs)

Beyond monitoring, new systems aim to provide portable renal replacement therapy [50,51]. Two notable examples include:

- AWAK (Automated Wearable Artificial Kidney): A peritoneal dialysis-based system that uses sorbent technology for dialysate regeneration, allowing for continuous, low-volume treatments [50,51].
- NEPHRON+: A prototype integrating sorbent regeneration and compact hemodialysis components, designed for wearable, extracorporeal use [50,51,52].

Impact: WAKs represent a transformative approach by offering continuous renal replacement in an ambulatory setting [50,51]. By reducing dependence on in-center dialysis, they increase patient autonomy, improve mobility, and potentially decrease cardiovascular stress associated with thrice-weekly dialysis sessions [50,51,52].

Metrics: Quality of life (QoL), patient mobility indices, ultrafiltration efficiency, biochemical clearance (urea, creatinine, phosphate), frequency of adverse events (e.g., clotting, device malfunction), and hospitalization rates [50,51,52].

### 2.5. Overall Significance

Wearables in nephrology, from simple monitoring devices to experimental artificial kidneys, are central to the shift toward personalized, decentralized kidney care [50]. While wearable sensors are already showing measurable clinical benefits, wearable dialysis systems remain at the experimental stage but hold promises to redefine freedom and survival for patients with ESRD [51,52].

#### 2.5.1. Artificial Intelligence and Predictive Analytics

The application of AI and predictive analytics is reshaping nephrology by enabling earlier risk detection, precision diagnostics, and individualized therapy [27,37]. These technologies leverage large-scale datasets from clinical, imaging, and biological sources to provide actionable insights that surpass traditional statistical models [53].

#### 2.5.2. AI for CKD Trajectory Prediction

Tools such as TrajVis have been developed to model and visualize CKD progression trajectories. These platforms integrate laboratory values with the estimated glomerular filtration rate (eGFR), proteinuria), demographics, and comorbidities to generate individualized risk predictions [54,55,56].

- Impact: Supports proactive, risk-based interventions, helping clinicians to identify patients who are likely to experience rapid decline and intensify therapy accordingly [54,55,56].
- Metrics: Prediction accuracy (Area Under the Curve (AUC)/(Receiver Operating Characteristic (ROC) values), calibration of risk scores, time gained for intervention, and reduction in rates of ESRD progression [54,55,56].

#### 2.5.3. AI Retinal Screening for CKD Detection

Systems such as RetiKid and RetiAge employ deep learning to analyze retinal images for microvascular changes linked to early kidney dysfunction. Retinal vasculature provides a non-invasive “window” for systemic microvascular health, correlating with early CKD [57,58].

- Impact: Enables large-scale, community-based CKD screening without invasive testing, which is particularly valuable in populations with limited access to nephrology services [57,58].
- Metrics: Sensitivity and specificity of early CKD detection, cost-effectiveness of screening programs, and number of cases identified at earlier disease stages [57,58].

#### 2.5.4. AI in Renal Pathology

Platforms including GloFinder, CellSpectra, and SISKA apply convolutional neural networks (CNNs) to kidney biopsies for automated detection of glomerular lesions, interstitial fibrosis, and other structural abnormalities [24,27,59]. Additionally, AI tools are being tested for drug stratification, identifying histologic features that are predictive of the therapeutic response [60,61].

- Impact: Increases reproducibility and speed of pathology assessment, reduces inter-observer variability, and enables biomarker discovery for precision medicine [24,27,59,60].
- Metrics: Diagnostic concordance with expert nephropathologists, turnaround time reduction, and accuracy of patient stratification for clinical trials [24,27,59,60].

#### 2.5.5. AI in Transplant Medicine

In transplantation, AI algorithms are being developed for non-invasive rejection detection (using biomarkers, histopathology, and imaging) and for guiding individualized immunosuppressive regimens [22,46,61,62,63]. Machine learning models integrate patient-specific variables (Human Leukocyte Antigens (HLA) mismatch, immunologic risk, drug levels, comorbidities) to optimize therapy while minimizing toxicity [59,62].

- Impact: Earlier detection of subclinical rejection, improved long-term graft survival, and safer immunosuppression management [59].
- Metrics: Time to rejection detection, graft survival rates, incidence of adverse drug effects, and cost-effectiveness compared to standard monitoring [59,62].

#### 2.5.6. Overall Impact of AI and Predictive Analytics in Nephrology

AI technologies offer unprecedented opportunities for personalized kidney care [56,59,60,62], with demonstrated potential to:

- Improve the prediction of CKD progression and optimize resource allocation.
- Enable early, non-invasive CKD detection through retinal imaging.
- Enhance diagnostic reproducibility and accelerate pathology workflows.
- Optimize transplant outcomes through predictive immunology.

Key metrics include prediction accuracy, time to clinical intervention, the cost-effectiveness of implementation, and long-term patient outcomes such as reduced CKD progression and improved graft survival [56,59].

AI tools may support earlier detection and risk stratification in CKD, although robust outcome data remain limited.

#### 2.5.7. Patient Engagement Apps and Self-Management

Digital health applications represent a critical bridge between healthcare providers and patients, enabling self-management and active engagement in CKD and related conditions [64,65]. By providing real-time tools for monitoring, reminders, and education, these apps empower patients to become co-managers of their disease [23,66].

#### 2.5.8. Utsarjan—A Pediatric Nephrology Application

Developed in India, Utsarjan is a mobile application that is specifically designed for children with nephrotic syndrome and their caregivers [49,67]. It enables the recording of symptoms (edema, urine protein status), monitoring of relapses, medication schedules, and communication with clinicians [68].

- Impact: Facilitates timely reporting of relapses, supports continuous follow-up, and enhances disease literacy among parents and children. In resource-limited settings, it helps to overcome barriers of distance and healthcare access [49,67].
- Metrics: Reduction in relapse-related hospitalizations, improved medication adherence rates, patient/caregiver satisfaction scores, and timely physician intervention [68].

#### 2.5.9. General Self-Management Apps in Nephrology

A growing ecosystem of kidney health apps provides functionalities such as [23,69,70]:

- Medication reminders (e.g., for phosphate binders, immunosuppressants, antihypertensive).
- Symptom tracking (blood pressure, weight, urine output, fatigue, pruritus).
- Dialysis logs to record ultrafiltration, session adherence, and dietary compliance.
- Educational content to improve disease awareness and empower behavioral change.

○ Impact: These tools reduce the communication gap between patients and providers, reinforce adherence to therapy, and encourage self-awareness of disease status [23]. They can also facilitate remote monitoring when integrated into clinical workflows [69].
○ Metrics: Percentage improvement in medication adherence, patient-reported outcome (PRO) measures (e.g., health-related quality of life, symptom burden), satisfaction scores, and reduced missed dialysis sessions [70].

#### 2.5.10. Overall Contribution

Patient engagement apps serve as low-cost, scalable interventions that complement formal telehealth platforms [68]. By enabling patients and families to track symptoms, adhere to medications, and communicate effectively with providers, these apps enhance self-efficacy and reduce preventable complications. In particular, pediatric-focused solutions such as Utsarjan highlight the potential of tailored digital tools to address the unique needs of vulnerable subpopulations [49,67,71].

#### 2.5.11. Data Dashboards and Clinical Decision Support

The increasing digitization of nephrology care has paved the way for data dashboards and clinical decision support systems (CDSS), which consolidate large volumes of clinical data into actionable insights [72,73,74]. These platforms enhance efficiency, reduce human error, and allow for earlier intervention through integrated risk prediction and trend visualization [72,74,75].

#### 2.5.12. Versia and AI-Enhanced Electronic Medical Records

Versia and similar EMR-integrated systems leverage advanced analytics to provide decision support directly at the point of care [56,76]. By consolidating the laboratory results, dialysis parameters, and patient-reported outcomes, these platforms generate automated alerts for high-risk patients (e.g., rapid eGFR decline, non-adherence, electrolyte abnormalities) [77]. Emerging AI-enhanced modules further improve predictive accuracy, offering personalized recommendations for therapy adjustment [78,79,80].

- Impact: Facilitates proactive interventions, reduces preventable adverse events, and standardizes the quality of care across dialysis centers [76].
- Metrics: Accuracy of decision-making (compared with guideline-based standards), reduction in adverse clinical events, and improved resource allocation efficiency [78,79,80].

#### 2.5.13. Analytics Dashboards (e.g., Sharesource Analytics 1.0)

The Sharesource Analytics 1.0 platform extends Vantive/Baxter’s RPM ecosystem by providing clinicians with aggregated dashboards for peritoneal dialysis patients [32,33]. They visualize adherence, catheter function, and longitudinal treatment trends, helping clinicians to identify the early warning signs of complications [42,54,72,81].

- Impact: Enhances the efficiency of patient monitoring by reducing manual data review, supports personalized prescription adjustments, and allows for rapid triage of patients requiring attention [32,33].
- Metrics: Reduction in missed alarms, earlier detection of catheter dysfunction, improved adherence tracking, and clinician workflow efficiency (time saved per patient review) [42,54].

#### 2.5.14. Overall Impact of Dashboards and CDSS

Together, data dashboards and AI-driven CDSS represent a shift toward evidence-informed, real-time decision-making in nephrology [27,46].

- They improve clinical efficiency by reducing the cognitive load on providers.
- They lower the risk of human error and oversight through automated alerts.
- They support standardization and quality improvement, ensuring consistent adherence to best practice.

Key metrics to evaluate their utility include decision accuracy, reduction in adverse events, workflow efficiency (time-to-decision, patient-to-provider ratio), and clinician satisfaction [27,46].

## 3. Discussion

ICT is reconfiguring nephrology by transforming how kidney care is delivered. Key advancements include:

- Remote management: Telehealth platforms and remote monitoring systems allow for continuous oversight of dialysis and transplant patients, reducing hospitalizations and improving adherence [8,14,17,33].
- Early detection: AI-driven predictive analytics, retinal screening, and wearable sensors enable proactive identification of disease progression and complications [27,58,67].
- Personalized treatment: Data dashboards, clinical decision support systems, and AI-enhanced EMRs facilitate individualized therapeutic strategies tailored to patient-specific risk profiles [56].
- Patient empowerment: Mobile apps, wearable devices, and self-management platforms engage patients in their care, enhancing adherence and health literacy [23,30,48,70,82].

The integration of AI, wearable technologies, telehealth, and centralized data platforms represents the next frontier of nephrology, offering the potential to shift care from reactive to proactive, precise, and patient-centered [83,84,85,86].

To fully realize these benefits, there is a pressing need for:

- Multicenter, long-term clinical trials to validate efficacy and safety.
- Cost-effectiveness analyses to support sustainable adoption across diverse healthcare settings.
- Equitable digital health strategies to bridge the digital divide and ensure access for all patient populations.

In summary, ICT tools are not merely adjuncts to traditional nephrology—they are defining the future of kidney care, fostering better outcomes, enhancing patient autonomy, and enabling a more proactive and personalized approach to managing CKD and ESRD [87].

ICT interventions and AI are the central drivers of the ongoing digital transformation in nephrology [27,46,88,89,90], enabling a shift from reactive care toward predictive, personalized, and data-driven clinical practice. As illustrated in Figure 2 (digital transformation model in nephrology), ICT and AI function as a core integrative layer that connects data acquisition, clinical analytics, decision support, and patient engagement across the kidney care continuum [14,19,23,25,26].

Within this model, AI algorithms leverage multimodal data derived from electronic health records, laboratory systems, imaging, wearable devices, and remote monitoring platforms to generate actionable clinical insights [26]. In chronic kidney disease (CKD), these capabilities support early disease detection, risk stratification, and prediction of progression trajectories [27]. By embedding AI within digital clinical workflows, nephrologists can identify high-risk patients earlier and tailor interventions more precisely, potentially delaying kidney failure and reducing healthcare utilization [29,30].

In dialysis care, the transformation model highlights the role of AI in continuous monitoring and adaptive treatment optimization. Predictive analytics enable the early identification of complications such as intradialytic hypotension, fluid imbalance, and vascular access dysfunction [78,80]. These AI-driven insights align with the feedback loops depicted in Figure 2, allowing for real-time clinical responses and iterative treatment refinement. Similarly, in transplantation, AI contributes to donor–recipient matching, graft survival prediction, and post-transplant surveillance, reinforcing the model’s emphasis on longitudinal and outcome-oriented care [8,30,91].

However, the implementation of AI within this digital transformation framework is accompanied by substantial challenges, as summarized in Table 1 (Challenges and Future Directions in Digital nephrology) [21,27]. Key barriers include data fragmentation, limited interoperability among digital systems, and concerns regarding data privacy and cybersecurity [92,93]. Additionally, the algorithmic bias and lack of model transparency raise ethical and clinical concerns, particularly when AI tools are deployed across diverse patient populations with varying socioeconomic and clinical characteristics [88,94].

From a clinical perspective, the integration of AI into routine nephrology practice also requires alignment with existing workflows and adequate clinician training [95]. Resistance to adoption may arise when AI systems are perceived as opaque or burdensome, rather than supportive [96]. As highlighted in Table 1, future directions must therefore prioritize explainable AI, user-centered design, and standardized validation methodologies to enhance trust and usability.

Although digital technologies show great promise for improving disease monitoring and supporting clinical decision-making, the data currently available remain heterogeneous, and the endpoints include mortality, hospitalization, or progression of CKD. The current evidence base is heterogeneous, and further prospective studies are needed to confirm their impact on patient-centered clinical outcomes.

Looking forward, prospective clinical trials and real-world implementation studies are essential to confirm the effectiveness, safety, and cost-efficiency of AI-driven interventions in nephrology [97]. The future digital nephrology ecosystem should emphasize ethical governance, regulatory oversight, and equitable access to ensure that AI-enabled innovations reduce—rather than exacerbate—healthcare disparities [98]. Integration of patient-generated data and shared decision-making tools will further strengthen the patient-centered dimension of the transformation model. ICT has shifted nephrology from reactive to proactive, bringing novelties such as improved outcomes, accessibility and patient empowerment.

Telemedicine interventions have shown potential to enhance care coordination and remote monitoring in nephrology, with some studies suggesting reductions in hospital utilization [97]. While many digital nephrology innovations demonstrate promising technical performance and feasibility, relatively few studies have evaluated their effects on hard clinical endpoints such as mortality, hospitalization, or progression of CKD, highlighting the need for well-designed prospective trials [99].

Further, high-quality evidence from RCT evaluation of long-term clinical outcomes remains limited.

### 3.1. Synthesis: From Reactive to Proactive Nephrology

The integration of ICTs has marked a fundamental transformation in nephrology, shifting care delivery from a traditionally reactive paradigm—focused on managing complications as they arise—to a proactive model, centered on prevention, prediction, and patient engagement [100,101].

Whereas conventional kidney care often relied on episodic, in-clinic assessments, ICT tools now enable continuous monitoring, early warning alerts, and individualized interventions [102]. Remote monitoring systems such as Sharesource and Versia, together with telenephrology platforms, have extended care beyond the dialysis center, enabling earlier detection of complications and reduced hospitalizations [32,33]. Similarly, wearables, AI-driven analytics, and patient engagement apps empower both clinicians and patients with real-time data to support timely decisions and self-management [23,26,46,82].

### 3.2. Strengths of ICT in Nephrology

1. Improved outcomes:

○ Remote monitoring and predictive analytics contribute to reduced hospitalization rates, better technique for survival in dialysis, and earlier detection of CKD progression or transplant rejection [27,37,60].
○ Continuous monitoring and decision support systems improve treatment precision and safety [65].

2. Accessibility:

○ Telehealth platforms extend specialist nephrology care to rural and underserved populations [5,15].
○ Mobile health applications enable scalable interventions, even in resource-limited settings [23,30,48].

3. Patient empowerment:

○ Engagement apps, wearable sensors, and educational platforms promote self-management, medication adherence, and active participation in care [70].
○ ICT tools strengthen communication channels between patients and providers, reducing gaps in follow-up and support [103].
○ In the case of the context of low- and middle-income countries (LMIC), telemedicine, portable diagnostics and remote patient monitoring can improve care for patients with CKD where nephrologists dialysis centers or imaging facilities are scarce [48,104].

### 3.3. Clinical Significance

By enabling earlier intervention, personalized care pathways, and enhanced patient engagement, ICT has shifted nephrology into an era of anticipatory medicine [27,58]. The emphasis is no longer on simply responding to complications, but on predicting, preventing, and empowering, thereby fostering more sustainable and patient-centered kidney care [67].

### 3.4. Challenges and Future Outlook in ICT for Nephrology

Future outlook: Precision nephrology, fully wearable kidneys, AI integration into daily practice, and Large Language Models (LLMs) for education and triage.

While the integration of ICT has accelerated innovation in nephrology, several challenges and barriers must be addressed before these technologies can be fully and equitably implemented.

### 3.5. Digital Divide: Access and Literacy

Not all patients have equal access to digital infrastructure. Limited internet connectivity, lack of affordable devices, and low digital literacy can exacerbate disparities in care. Older adults and socioeconomically disadvantaged groups are particularly at risk of exclusion from ICT-enabled kidney care [12,19,105]. Bridging this divide requires targeted policies, training programs, and community-based digital health initiatives.

### 3.6. Data Privacy, Security, and Interoperability

ICT platforms generate large volumes of sensitive health data. Ensuring data privacy, cybersecurity, and interoperability across multiple electronic health record (EHR) systems remains a major challenge [93]. Without common standards, data silos hinder seamless integration of wearable data, telehealth records, and AI analytics into unified care workflows [12].

### 3.7. Cost and Reimbursement

The financial sustainability of ICT-driven kidney care depends on clear reimbursement models. While pilot programs show clinical benefit, lack of structured payment frameworks and uncertainties around insurance coverage limit scalability. Initial investments in infrastructure and training may also be prohibitive for smaller dialysis centers or health systems in low-resource settings [106,107].

### 3.8. Need for Regulatory Validation and Randomized Trials

Many ICT tools remain at the prototype or pilot stage, with limited validation through RCTs [23,24,43,45,53]. Regulatory bodies require robust evidence on safety, efficacy, and cost-effectiveness before approving large-scale adoption. Without rigorous validation, the risk of overpromising benefits may undermine trust among clinicians and patients [59,60].

In nephrology research, an RCT might evaluate whether a digital health intervention improves outcomes in patients with CKD. The evidence from RTC is often considered to be high-level clinical evidence because it reduces confounding factors, minimizes selection bias, and allows for stronger conclusions about cause-and-effect relationships.

Researchers then compare outcomes such as hospitalization rates, disease progression, or treatment adherence [23].

For this reason, many guidelines and regulatory decisions rely heavily on RCT evidence when evaluating new medical treatments, technologies, or clinical interventions [23,106].

### 3.9. Comparison with Traditional Care

ICT-enabled care must be critically compared with conventional nephrology models. While remote monitoring and AI can reduce hospitalizations and improve adherence, concerns remain about reduced face-to-face interactions, potential depersonalization of care, and reliance on algorithms that may not fully capture patient complexity. Hybrid models combining digital and traditional approaches are likely to be the most effective [13,35,36,44].

### 3.10. Future Outlook

Despite these challenges, the future of ICT in nephrology is promising [19,20,96], with several trajectories gaining momentum:

- Precision nephrology: Integration of multi-omics, imaging, and AI-driven analytics will enable personalized treatment plans tailored to individual disease biology [56,65].
- Fully wearable kidneys: Advances in sorbent regeneration and miniaturization technologies may allow for continuous, portable dialysis, dramatically improving autonomy and quality of life [31].

In this context, innovative approaches to dialysis are being explored. Although frequent or daily dialysis has demonstrated clinical benefits, its widespread implementation remains challenging, due to logistical and lifestyle constraints. Emerging technologies, such as the WAK, represent a promising alternative, enabling more frequent dialysis, minimizing disruption to patients’ daily activities, and potentially improving their quality of life. However, these systems remain under clinical investigation, and their impact on long-term patient outcomes has not yet been fully established. The feasibility of such systems depends on the successful integration of key dialysis components into a portable device, including dialysis membranes, dialysate regeneration systems, vascular access, patient monitoring technologies, and reliable power sources. Among these, efficient blood and dialysate pumping systems remain critical technical challenges in the development of wearable dialysis platforms [6].

- AI integration into daily practice: Predictive analytics, pathology AI, and real-time decision support will become standard components of nephrology workflows [38,108].
- Large Language Models (LLMs): Generative AI systems can provide clinical documentation support, patient triage, and education, potentially reducing clinician workload while improving patient understanding [28,99].

### 3.11. Overall Perspective

Addressing the barriers of access, equity, and validation is essential to realize the full potential of ICT in nephrology. If these challenges are systematically addressed [43,55], ICT tools will not merely complement traditional nephrology but reshape the field into a predictive, personalized, and patient-empowered discipline [27,37,60].

The implementation of advanced digital and extracorporeal platforms in nephrology must take into account the growing complexity of modern healthcare systems and patient populations. In the treatment of acute kidney injury (AKI), patients may require extracorporeal blood purification therapy (EBPT), which involves several interconnected components and high-risk clinical workflows. These systems increase the potential for organizational and process-related errors if not carefully managed. Therefore, their safe implementation requires structured clinical risk management strategies, including standardized protocols, operating procedures, and checklists. Multidisciplinary collaboration, staff training, and clear communication channels are also essential to ensure safe device operation and proactive identification of potential system vulnerabilities in complex extracorporeal care settings [109].

## 4. Limitations

### 4.1. Limited Long-Term Evidence

Many ICT tools, including remote monitoring platforms, wearable sensors, and AI-based predictive models, have primarily been evaluated in short-term or pilot studies. Evidence demonstrating the sustained impact on clinical outcomes such as long-term graft survival, CKD progression, or patient-reported quality of life remains limited. Without long-term follow-up data, it is challenging to fully quantify the benefits, potential risks, or cost-effectiveness [8,106,110].

The level of evidence supporting digital technologies in nephrology varies substantially across different applications or interventions. Consequently, caution is warranted when interpreting reported benefits, as stronger evidence from large multicenter studies and RCT is still required to confirm their effectiveness in routine nephrology practice [23,106].

### 4.2. Publication Bias

The current literature is heavily skewed toward studies conducted in high-income countries, where access to digital infrastructure and healthcare resources is greater. This introduces publication bias and may limit the generalizability of findings to low- and middle-income settings, where challenges such as digital literacy, connectivity, and healthcare infrastructure are more pronounced [21,51].

### 4.3. Variability in Regulatory Approvals

ICT tools for nephrology often span multiple regulatory categories, including medical devices, software-as-a-medical-device (SaMD), and AI-driven algorithms [27]. Regulatory requirements vary across regions, creating heterogeneity in approval, adoption, and clinical integration [12]. This variability complicates the large-scale deployment and standardization of digital kidney care.

### 4.4. Limitations of the Current Evidence Base and Future Research Needs

Recognizing these limitations is essential for interpreting currently available data and for designing robust future studies, including multicenter trials and longitudinal evaluations that address existing gaps in long-term data. Reducing publication bias and harmonizing regulatory procedures will be essential to ensuring that ICT-based nephrological interventions are safe, effective, and globally applicable.

## 5. Comparison with Existing Reviews and Novel Contribution of This Review

Several recent reviews have explored the integration of digital technologies within nephrology, including telemedicine applications, AI in imaging and prediction models, and remote monitoring systems [49]. However, most available reviews focus on single technological domains (e.g., telehealth platforms, AI-based diagnostics, or wearable dialysis technologies), rather than examining their system-level integration across the nephrology care continuum [44,103].

For example, previous reviews have examined the role of AI in nephrology diagnostics, particularly deep learning methods such as Convolutional Neural Networks when applied to imaging analysis, including renal ultrasound and histopathology interpretation. Similarly, other studies have evaluated telemedicine implementation in chronic kidney disease (CKD) management and the feasibility of remote patient monitoring programs for dialysis patients. Reviews addressing wearable technologies have largely concentrated on the engineering aspects of devices such as the wearable artificial kidney, with limited discussion on their integration into clinical workflows or healthcare systems [50].

Despite these contributions, the literature remains fragmented in three key aspects:

- Lack of ecosystem perspective:
  Existing reviews rarely conceptualize digital nephrology as a multi-layered technological ecosystem integrating AI, telehealth, imaging, wearable monitoring, and data platforms.
- Limited focus on clinical workflow integration:
  Many technological studies evaluate performance metrics (e.g., the predictive accuracy of AI models) but provide limited discussion on clinical implementation pathways, workflow redesign, and interoperability within health systems.
- Underrepresentation of safety and process-design considerations:
  Few reviews address the organizational, operational, and safety challenges associated with deploying complex extracorporeal platforms and digital monitoring systems in critical care nephrology.

The current literature largely reflects early-phase evidence focused on feasibility, technical validation, and predictive performance, whereas the robust clinical outcome data remain limited.

### 5.1. Novel Contribution of This Narrative Review

The present review seeks to address these gaps by providing a comprehensive systems-level framework for digital nephrology, integrating emerging technologies across diagnostic, monitoring, and therapeutic domains. Specifically, this manuscript contributes to the literature by:

- Conceptualizing a digital nephrology ecosystem, integrating ICT, AI-driven diagnostics, wearable sensors, telehealth platforms, and remote monitoring tools across the CKD and dialysis continuum.
- Linking technological innovation with clinical workflow redesign, highlighting ultrasound-enabled workflows, AI-assisted decision support, and digital vascular access monitoring strategies.
- Addressing implementation barriers and safety considerations, including the organizational risk management, protocol standardization, and process-design strategies required for complex extracorporeal therapies.
- Integrating patient-centered technological innovation, including wearable dialysis systems and mobile health platforms designed to improve quality of life and treatment adherence.

By combining these elements, the review provides a translational framework bridging technological innovation and clinical nephrology practice, thereby expanding the current understanding of how digital health tools can support the management of chronic kidney disease and renal replacement therapies.

### 5.2. Future Directions and Research Agenda

The rapid evolution of digital technologies is expected to reshape nephrology practice across diagnostic, monitoring, and therapeutic domains. Future research should prioritize the development of integrated digital nephrology ecosystems that are capable of combining AI, remote monitoring, imaging platforms, and wearable technologies into interoperable clinical workflows. In particular, machine learning approaches such as convolutional neural networks (CNN) and other deep learning architectures may enhance diagnostic accuracy in kidney imaging, risk prediction of disease progression, and early detection of complications in patients with CKD and AKI.

A critical priority for the next generation of studies is the transition from proof-of-concept technologies to rigorous clinical validation. Prospective multicenter trials should evaluate the clinical effectiveness, safety, and cost-effectiveness of digital health platforms in nephrology care pathways. In parallel, pragmatic implementation studies are needed to assess the integration of telehealth systems, AI-assisted ultrasound workflows, and remote patient monitoring into routine clinical practice. Artificial intelligence algorithms, including deep learning models such as CNN, have demonstrated promising diagnostic performance in kidney imaging and prediction. However, further studies are needed to determine whether these improvements translate into measurable clinical outcome benefits.

Technological innovation should also focus on patient-centered solutions, including wearable dialysis systems and continuous physiological monitoring devices. Advances in portable extracorporeal technologies, such as the wearable artificial kidney, may enable more flexible and frequent renal replacement therapies while reducing the burden associated with conventional dialysis.

Finally, future research must address organizational, regulatory, and ethical challenges, including data interoperability, cybersecurity, algorithm transparency, and clinical governance frameworks. Addressing these dimensions will be essential to ensure that digital nephrology technologies translate into safe, equitable, and sustainable improvements in kidney care.

Future progress will depend on robust clinical validation and integration into routine care pathways, and also include precision nephrology, fully wearable kidneys, AI integration, and large language models for education and triage.

## 6. Conclusions

In conclusion, when viewed through the lens of the digital transformation model in nephrology, AI emerges as a foundational enabler of precision kidney care [56,65]. Addressing the challenges and future directions outlined in Table 2 will be critical for translating AI from experimental innovation into sustainable clinical practice. Thoughtful implementation of AI within a comprehensive digital framework holds substantial promise for improving kidney health outcomes and redefining the future of nephrology [8,30].

Digital nephrology represents more than technological innovation—it is a redefinition of how kidney disease is managed. ICT tools are transforming patient–provider relationships, enabling precision interventions, and extending care to new contexts. The revolution, however, will only be realized if technological progress is matched with ethical governance, equitable access, and system-level integration.

By aligning technological innovation with clinical needs and health system capacity, ICT can play a pivotal role in transforming kidney care for the future [108]. ICT has reshaped nephrology, calling for integration, validation, and equitable access. The future of nephrology lies at the intersection of digital innovation and human-centered care.

Digital technologies may support improved disease monitoring and clinical decision-making in patients with kidney disorders and those receiving RRT, although robust evidence demonstrating direct improvements in clinical outcomes remains limited.

Challenges include digital divide, regulatory heterogeneity, cost, and the need for long-term evidence.

## Figures and Tables

**Table 1:** Inclusion and exclusion criteria.

Inclusion Criteria	Exclusion Criteria
The study focuses on AI applications and ICT intervention in kidney care, including diagnostics, treatment, prevention, efficiency, and processes innovation.	Irrelevant clinical focus: Studies that did not focus on AI and/or ICT intervention in nephrology were not directly related to digital nephrology.
The study must discuss the application of “artificial intelligence”; “machine learning (ML)”; “ICT interventions”; “diagnostics”; “treatment”; “prevention”; “predictive modeling”; telemedicine and telemonitoring platforms; digital imaging workflows; remote patient monitoring systems; digital clinical decision support systems; and “wearable technology” in nephrology.	Articles with limited access, reports, doctoral theses and duplicate publications were excluded.
Peer-reviewed articles, systematic reviews, scoping reviews, and narrative reviews providing relevant conceptual insights are included.	Non-peer-reviewed articles or studies lacking experimental data or relevant reviews. Articles with insufficient methodological information to access the relevance.
Wearable technologies used in the nephrology field.	
Included studies have been published in the English language from 2011 to 2025.	Excluded studies published in non-English languages.
Studies involving adult or pediatric patients (human) with kidney disorders.	Non-human studies.

**Table 2:** ICT tools driving digital nephrology.

ICT Tool	Function in Nephrology	Example Use Case	Transformative Impact
Telenephrology	Virtual consultations	Dialysis follow-up and transplant care	Expands care beyond hospitals
Mobile Health Applications (Apps)	Self-management support	Medication, diet, and fluid monitoring	Empowers patient engagement
Artificial Intelligence/Machine Learning	Predictive analytics	AKI risk prediction, transplant rejection alerts	Shifts from reactive to proactive care
Wearable Technologies	Continuous monitoring	BP, hydration, and dialysis adequacy	Enables real-time interventions
EHR (Electronic Health Record) Integration	Interoperability & data sharing	Unified nephrology records	Enhances care coordination

**Table 3:** Main use-case, function and evidence level of ICT tools developed to be used in nephrology and kidney care.

Classification/Maturity Level	ICT Tool/Example	Main Use-Case/Function	Evidence Level (Short)
Proven	Remote patient management platforms (e.g., Sharesource/Claria/HomeChoice Claria)	Continuous monitoring of peritoneal dialysis (PD) exchanges, adherence, alerts for technique/failure.	Multiple clinical evaluations, registry/presenter data show improved adherence, reduced technique failure and earlier intervention.
Proven	Telehealth/video visits (telenephrology)	Routine outpatient follow-up, triage, education, post-transplant and dialysis consults.	Systematic reviews and multiple clinical series show improved access, patient satisfaction and reduced travel; established during COVID-19.
Proven	EHR-integrated dashboards & registries	Population management, CKD registries, risk stratification, referrals.	Proven value for care coordination and audit; many centers use dashboards to track labs and dialysis metrics.
Emerging	AI-based risk prediction (CKD progression models)	Predict who will progress, personalize follow-up frequency and therapy.	Rapidly growing literature with promising retrospective and some prospective validations; need external validation and bias audits.
Emerging	AI for pathology/biopsy image analysis	Automated quantification of fibrosis, glomerular lesion detection.	Multiple proof-of-concept and validation studies show improved speed/accuracy vs. manual scoring in some tasks.
Emerging	Connected dialysis machines & telemonitoring for HD (e.g., Versia)	Remote monitoring of HD session metrics, alarms, machine telemetry.	Growing pilot deployments; mixed but improving evidence; vendor pilots and conference data.
Emerging	Wearables & home sensors (BP, weight, fluid status, activity trackers)	Home physiologic monitoring to detect fluid overload, BP control, activity.	Pilot trials show feasibility; growing integration with mobile apps; evidence for clinical outcome improvement is emerging.
Experimental	Edge/fog computing, blockchain for secure RPM	Low-latency processing, immutable audit trails for device data.	Mostly proof-of-concept and engineering papers; not broadly used clinically in nephrology yet.
Experimental	Predictive closed-loop dialysis prescription (auto titration using ML)	Automated prescription adjustments based on continuous data.	Early experimental systems and small pilots; no widespread clinical adoption yet.
Experimental	Generative AI for clinical documentation and patient education	Drafting notes, summarizing encounters, generating patient-facing educational material.	Early generation tools tested; accuracy, hallucination risk and privacy concerns require caution.
Experimental	Implantable sensors for continuous fluid/pressure monitoring	Direct continuous monitoring of intravascular volume or renal hemodynamics.	Preclinical/very early human feasibility studies only.

Proven technologies: Technologies with strong clinical validation and established use in routine care. Emerging technologies: Technologies showing promising results but still undergoing clinical validation. Experimental technologies: Technologies in early development stages or proof-of-concept testing.

**Table 4:** Example vendor/study and implementation note of ICT tools developed to be used in nephrology and kidney care.

Classification/Maturity Level	ICT Tool/Example	Example Vendor /Study	Implementation Note
Proven	Remote patient management platforms (e.g., Sharesource/Claria/HomeChoice Claria)	Vantive/Baxter Sharesource/Claria; published single-center and multicenter evaluations.	Widely used in APD programs, it requires integration with clinic workflows and staff to act on alerts.
Proven	Telehealth/video visits (Telenephrology)	Many general telehealth vendors and nephrology programs; several scoping reviews/case series.	Low barrier to adopt; needs clinical protocols, data privacy safeguards, and reimbursement pathways.
Proven	EHR-integrated dashboards & registries	Local/regional EHR tools, national registries (varies by country).	Integration & interoperability are common barriers; human-centered design improves uptake.
Emerging	AI-based risk prediction (CKD progression models)	Academic groups and vendor solutions; reviewers highlight potential but call for validation.	Strong potential to inform triage; must meet regulatory/validation standards before routine use.
Emerging	AI for pathology/biopsy image analysis	CNN/CV models from research groups; early-commercial partnerships emerging.	Useful for workload reduction and standardization; needs multi-center validation.
Emerging	Connected dialysis machines & telemonitoring for HD (e.g., Versia)	Vendor solutions (various dialysis manufacturers, e.g., Vantive/Baxter).	Requires robust networking, cybersecurity and clinical response pathways.
Emerging	Wearables & home sensors (BP, weight, fluid status, activity trackers)	Consumer-grade and medical-grade devices integrated via platforms.	Patient adherence and data validation are common challenges.
Experimental	Edge/fog computing, blockchain for secure RPM	Academic/industry prototypes.	Promising for security and offline resilience; complexity and regulation remain issues.
Experimental	Predictive closed-loop dialysis prescription (auto titration using ML)	Research prototypes, limited pilots.	Safety, regulatory approval and clinician trust are major hurdles.
Experimental	Generative AI for clinical documentation and patient education	LLMs/vendor experiments; early evaluations in nephrology.	Useful for efficiency but must be supervised and validated for clinical accuracy.
Experimental	Implantable sensors for continuous fluid/pressure monitoring	Academic/industry.	

Proven technologies: Technologies with strong clinical validation and established use in routine care. Emerging technologies: Technologies showing promising results but still undergoing clinical validation. Experimental technologies: Technologies in early development stages or proof-of-concept testing. CNN: Convolutional neural networks. CV: Computer vision.

**Table 5:** Function and clinical impact of ICT tools that are currently reconfiguring nephrology and kidney care.

Category	Tool/Example	Function	Clinical Impact
Remote Monitoring & Telehealth	Sharesource (Vantivbe/Baxter)	Remote patient management for automated peritoneal dialysis (APD).	Enables bidirectional monitoring, earlier intervention, reduced complications.
Data Collection and Storage	Versia (Vantive/Baxter)	Renal data management system.	Centralizes dialysis/patient data, supports decision-making & workflow.
Home Monitoring	MACCS platform (Germany)	Digital home monitoring for kidney transplant patients.	Patient self-reporting (vitals, meds, labs), integrated with transplant centers.
Telemedicine	Telenephrology platforms	Virtual consultations & follow-up.	Expands specialist access, continuity of care, reduces travel.
Wearables & Portable Dialysis	NEPHRON+ project	Wearable artificial kidney and remote monitoring.	Enables ambulatory dialysis with multiparametric sensors.
Patient Mobility	AWAK/portable PD devices	Sorbent-based regenerative dialysis.	Mobility, reduced dialysate burden.
	Wearable sensors (BP, hydration, glucose)	Continuous patient physiology monitoring.	Early detection of fluid overload, hypertension.
Artificial Intelligence & Predictive Analytics	TrajVis	CKD trajectory visualization with AI predictions.	Supports early intervention, personalized therapy.
AI for Screening	RetiKid/RetiAge	AI retinal imaging for CKD risk screening.	Non-invasive early detection in primary care.
AI for Diagnostics	AI pathology tools (CellSpectra, SISKA, GloFinder)	Automated histopathology & cell stratification.	Improves biopsy analysis, guides targeted therapy.
Artificial Intelligence & Predictive Analytics	AI in transplantation	Risk prediction for rejection, immunosuppression guidance.	Prevents graft loss, optimizes therapy.
Patient Engagement & Apps	Utsarjan (India)	Mobile app for nephrotic syndrome in children.	Supports medication tracking, labs, real-time guidance.
Reminder & Communication	Dialysis log & reminder apps	Patient self-monitoring tools.	Enhances adherence, reduces missed sessions.
Data Dashboards & Clinical Decision Support	Sharesource Analytics 1.0	Trend analysis of APD treatment data.	Identifies early issues (catheter function, adherence).
AI for Predictive Alerts	AI-enhanced EMRs	Integrates labs, dialysis data, predictive alerts.	Improves safety, clinician efficiency.

**Table 6:** Key outcomes and maturity level of ICT tools that are currently reconfiguring nephrology and kidney care.

Category	Tool/Example	Key Outcomes Measured	Maturity Level
Remote Monitoring & Telehealth	Sharesource (Vantivbe/Baxter)	Technique survival, hospitalization rate, adherence, patient satisfaction.	Proven
Data collection and storage	Versia (Vantive/Baxter)	Data completeness, decision accuracy, workflow efficiency.	Proven/early adoption
Home Monitoring	MACCS platform (Germany)	Medication adherence, graft function, adverse events.	Proven (pilot studies)
Telemedicine	Telenephrology platforms	Patient access, visit frequency, satisfaction, cost savings.	Proven
Wearables & Portable Dialysis	NEPHRON+ project	Fluid balance, BP control, QoL, device safety.	Experimental
Patient Mobility	AWAK/portable PD devices	Dialysate volume reduction, patient mobility, QoL.	Experimental
	Wearable sensors (BP, hydration, glucose)	BP trends, interdialytic weight gain, symptom detection.	Emerging
Artificial Intelligence & Predictive Analytics	TrajVis	Prediction accuracy, CKD progression, time-to-intervention.	Emerging
AI for Screening	RetiKid/RetiAge	Screening sensitivity/specificity, early CKD diagnosis.	Emerging
AI for Diagnostics	AI pathology tools (CellSpectra, SISKA, GloFinder)	Lesion detection accuracy, treatment stratification.	Emerging
Artificial Intelligence & Predictive Analytics	AI in transplantation	Graft survival, immunosuppression dosing accuracy.	Experimental/emerging
Patient Engagement & Apps	Utsarjan (India)	Adherence %, relapse detection, parent/patient satisfaction.	Emerging
Reminder & Communication	Dialysis log & reminder apps	Adherence rates, QoL, communication with care team.	Emerging
Data Dashboards & Clinical Decision Support	Sharesource Analytics 1.0	Event detection rate, intervention timeliness.	Proven
AI for Predictive Alerts	AI-enhanced EMRs	Reduced adverse events, clinician time saved.	Emerging

Proven technologies: Technologies with strong clinical validation and established use in routine care. Emerging technologies: Technologies showing promising results but still undergoing clinical validation. Experimental technologies: Technologies in early development stages or proof-of-concept testing.

## Data Availability

No new data were created or analyzed in this study.

## Abbreviations

AI	Artificial Intelligence
AKI	Acute Kidney Injury
APD	Automated Peritoneal Dialysis
APPs	Applications
AUC	Area Under the Curve
AWAKs	Automated Wearable Artificial Kidney
BP	Blood Pressure
CDSS	Clinical Decision Support Systems
CKD	Chronic Kidney Disease
CGM	Continuous Glucose Monitoring
CNN	Convolutional Neural Network
CV	Computer Vision
EBPT	Extracorporeal Blood Purification Treatment
eGFR	Estimated Glomerular Filtration Rate
EHR	Electronic Health Record
ESRD	End-Stage Renal Disease
HLA	Human Leukocyte Antigens
HD	Hemodialysis
ICT	Information and Communication Technologies
KRT	Kidney Replacement Therapy
LLMs	Large Language Models
LMIC	Low-and Middle-Income Countries
MACCS	Medical Assistant for Chronic Care Service/Support
ML	Machine Learning
PD	Peritoneal Dialysis
PRO	Patient Reported Outcome
QoL	Quality of Life
RCTs	Randomized Controlled Trial
RM	Remote Monitoring
ROC	Receiver Operating Characteristic
RPM	Remote Patient Monitoring
RRT	Renal Replacement Therapy
SaMD	Software as a Medical Device
WAK	Wearable Artificial Kidney
